# Re: Jones et al., *Nutrients* 2018, *10*, 501

**DOI:** 10.3390/nu10060746

**Published:** 2018-06-09

**Authors:** Mark Lawrence, Julie Woods

**Affiliations:** Institute for Physical Activity and Nutrition (IPAN), School of Exercise and Nutrition Sciences, Deakin University, Geelong 3220, Australia; j.woods@deakin.edu.au

Dear Editor,

We read with interest the paper by Jones et al. [1] that concludes there is “good” alignment between the Australian Dietary Guidelines (ADGs) [2] and the Health Star Rating (HSR) System [3]. The study also asserts that where misalignment arises, this is due to specific food product “outliers”, the existence of which is overwhelmingly attributed to failures in the ADGs. However, the conclusion and assertion are incorrect because they significantly underestimate the extent of, and incorrectly attribute cause for, the misalignment between the ADGs and HSR. These errors are a consequence particularly of two scientific flaws: flawed assessment against nutrition science classifications; and flawed interpretation of nutrition science evidence.

## 1. Flawed Assessment against Nutrition Science Classifications 

The researchers arbitrarily set the HSR cut-off points used for assessing whether a food is ‘healthy’ or not and whether a discretionary or core food is ‘apparently’ misclassified in terms of the HSR. For instance, discretionary foods were assessed as outliers if they displayed a HSR ≥ 3.5; the reasoning behind this liberal cut-off value was not provided. What this meant in the assessment process was that the 4156 discretionary foods recorded as displaying 2.5 or 3 ‘health’ stars, including: ice creams and frozen ice confections; jellies; chocolate chip muesli bars; and party pies were not considered outliers, instead they were all assessed as aligning with ADG recommendations. These star ratings (2.5 and 3), which essentially constitute at least a ‘pass’ mark for discretionary foods are inconsistent with the ADGs which advise that discretionary foods “are not a necessary part of the diet” and most Australians consume too many of them [2]. 

The result of this flawed assessment is that the level of misalignment between the HSR and ADGs was significantly underestimated. Previously we have reported that 57% of new discretionary foods entering the marketplace since the commencement of the HSR system display ≥2.5 stars and concluded that, in its current form, the HSR is undermining the ADGs [4]. As with the Jones et al. study the cut-off point used in our study was arbitrary. However, unlike the Jones et al. study, the ≥2.5 stars point that we set as a cut off for assessing discretionary food misalignment was reasoned. The reasoning being that if the evidence shows that most Australians need to eat less discretionary foods then it is incongruous in policy practice to accept a rating of ≥2.5 stars out of 5, i.e., a pass, for any of these food products. Unfortunately the Jones et al. paper misrepresents our assessment of the extent of the misalignment by asserting that we simply highlighted the existence of some “high profile products”. This is incorrect. We demonstrated the misalignment is systemic and a consequence of inherent design flaws in the current HSR system. 

Our study and the Jones et al. study drew data from different databases with contrasting strengths and weaknesses. The database we used records what is actually happening in the marketplace in terms of foods available and HSR uptake on an almost daily basis, an important capability when assessing rapid food product and labelling innovation responses to food policies and regulations, but it only collects information on new food products. The database used by Jones et al. is based on cross sectional, annual surveys and relies on the HSR being calculated from the nutrition information panel (with statistical adjustment required for missing data), but it is large and reflects new and existing foods in the marketplace. Yet these database differences do not explain the substantial difference in the level of misalignment recorded by the two studies, because the HSR uptake rate and profile of all new foods assessed in our study was comparable with the uptake rate and profile for combined existing and new foods recorded in other surveys [5].

The flawed assessment extended to whether a discretionary or core food is misclassified in terms of the HSR. This aspect of the assessment process involved subjecting discretionary foods receiving a HSR ≥ 3.5 and initially classified as outliers to a secondary assessment according to traffic light criteria for salt, total sugars and/or saturated fat content [6]. If these products did not receive a red traffic light for any of these nutrients they were discounted as being true outliers and it was concluded that the correct HSR had been applied. For instance, there were 173 apparent confectionary outliers but 161 of these products did not score a red traffic light for salt, total sugar or saturated fat and so they were reasoned to be consistent with the ADGs. The authors suggest that this signals a ‘failure’ of the ADGs because, presumably, the ADGs shouldn’t be classifying some confectionary as discretionary. It is concerning then to see this type of flawed reasoning applied to the 4105 foods initially classified as discretionary (scoring a HSR ≥ 3.5) to claim that 3130 were erroneously classified by the ADGs. 

## 2. Flawed Interpretation of Nutrition Science Evidence 

The development of the ADGs and HSR reflect contrasting nutrition science approaches to evidence synthesis and translation. The ADGs were informed by robust evidence synthesis and translation protocols and procedures including steps to assess and then manage the potential for conflicts of interest. Evidence was synthesised from five sources, such as the findings of a series of systematic reviews of 55,000 studies. The synthesised evidence was translated into the ADGs through the application of a 2-step rigorous procedure whereby the quality of the evidence and the strength of the recommendation were assessed against reference standards. 

By contrast, there was no formal evidence synthesis and translation procedure to inform the HSR. Instead a group of experts representing food manufacturers, government representatives and public health/consumer advocacy organisations agreed upon a limited selection of nutrients and then set arbitrary cut-off points to include in an algorithm to calculate the number of stars to allocate to a food. There is an absence of any record of how potential conflicts of interest were assessed or managed.

The result of this flawed interpretation of nutrition science evidence is an incorrect causal attribution of the misalignment in terms of its assertion that the problem is due predominantly to the ADGs and not the HSR. It is the ADGs that are informed by robust scientific evidence and not the HSR. Effectively the researchers are elevating a procedure relying on expert opinion and lacking a formal conflict of interest assessment above rigorous scientific procedures complemented with conflict of interest risk assessment and management as the preferred basis for informing food and nutrition policy.

We agree with the Jones et al.’s study regarding the need for a review of the algorithm for points awarded for added sugars rather than total sugars and for salt. Also food manufacturers must be prevented from gaming the algorithm by adding fibre and protein ingredients to garner extra points. But the causes of misalignments are more fundamental than just technical faults with the algorithm. Primarily they are due to a design flaw in the HSR system. The HSR system design urgently needs reform by capping the maximum number of stars available to a discretionary food to below a ‘pass’ or replacing stars with warning symbols of the type being implemented in countries such as Chile. Only then should the HSR be mandated, otherwise such a development would be premature for it would only serve to increase the prevalence of incorrect HSRs in the marketplace. 

In its current form, the HSR is undermining the ADGs by providing a vehicle for promoting discretionary foods. Unfortunately its systemic faults are being covered up by flawed research of the type reported in this paper. The result is that the inaccurate findings are misleading decision-makers about the HSR performance, disparaging the ADGs and serving inadvertently to embolden junk food marketing.
